# Economic Costs and Health-Related Quality of Life Outcomes of HIV Treatment After Self- and Facility-Based HIV Testing in a Cluster Randomized Trial

**DOI:** 10.1097/QAI.0000000000001373

**Published:** 2017-06-14

**Authors:** Hendramoorthy Maheswaran, Stavros Petrou, Peter MacPherson, Felistas Kumwenda, David G. Lalloo, Elizabeth L. Corbett, Aileen Clarke

**Affiliations:** * Division of Health Sciences, University of Warwick Medical School, Coventry, United Kingdom;; † Malawi-Liverpool-Wellcome Trust Clinical Research Programme, Blantyre, Malawi;; ‡ Department of Public Health and Policy, University of Liverpool, Liverpool, United Kingdom;; § Department of Clinical Sciences, Liverpool School of Tropical Medicine, Liverpool, United Kingdom; and; ‖ London School of Hygiene and Tropical Medicine, London, United Kingdom.

**Keywords:** HIV, HIV self-testing, ART, costs, health-related quality of life, EQ-5D

## Abstract

The scale-up of HIV self-testing (HIVST) in Africa is recommended, but little is known about how this novel approach influences economic outcomes following subsequent antiretroviral treatment (ART) compared with established facility-based HIV testing and counseling (HTC) approaches.

**Setting:**

HIV clinics in Blantyre, Malawi.

**Methods:**

Consecutive HIV-positive participants, diagnosed by HIVST or facility-based HTC as part of a community cluster-randomized trial (ISRCTN02004005), were followed from initial assessment for ART until 1-year postinitiation. Healthcare resource use was prospectively measured, and primary costing studies undertaken to estimate total health provider costs. Participants were interviewed to establish direct nonmedical and indirect costs over the first year of ART. Costs were adjusted to 2014 US$ and INT$. Health-related quality of life was measured using the EuroQol EQ-5D at each clinic visit. Multivariable analyses estimated predictors of economic outcomes.

**Results:**

Of 325 participants attending HIV clinics for assessment for ART, 265 were identified through facility-based HTC, and 60 through HIVST; 168/265 (69.2%) and 36/60 (60.0%), respectively, met national ART eligibility criteria and initiated treatment. The mean total health provider assessment costs for ART initiation were US$22.79 (SE: 0.56) and US$19.92 (SE: 0.77) for facility-based HTC and HIVST participants, respectively, and was US$2.87 (bootstrap 95% CI: US$1.01 to US$4.73) lower for the HIVST group. The mean total health provider costs for the first year of ART were US$168.65 (SE: 2.02) and US$164.66 (SE: 4.21) for facility-based HTC and HIVST participants, respectively, and comparable between the 2 groups (bootstrap 95% CI: −US$12.38 to US$4.39). EQ-5D utility scores immediately before and one year after ART initiation were comparable between the 2 groups. EQ-5D utility scores 1 year after ART initiation had increased by 0.129 (SE: 0.011) and 0.139 (SE: 0.027) for facility-based HTC and HIVST participants, respectively.

**Conclusions:**

Once HIV self-testers are linked into HIV services, their economic outcomes are comparable to those linking to services after facility-based HTC.

## Abstract 2

Supplemental Digital Content is Available in the Text.

## INTRODUCTION

There are now over 10 million Africans receiving antiretroviral treatment (ART), the majority living in Eastern and Southern Africa.^1^ Despite this impressive achievement, over one half of HIV-positive individuals are still in need of treatment, and over one million people become infected every year.^1^ Meeting HIV elimination targets set by UNAIDS (“90-90-90”) will require novel approaches and significant investment in HIV testing and treatment services. HIV self-testing (HIVST), defined as an individual performing and interpreting their own HIV test,^2^ is one potential solution, and its scale-up in Africa is recommended.^3^

HIVST offers an opportunity for early engagement of individuals in HIV services.^4,5^ However, there is limited research around the cost implications and health-related quality of life (HRQoL) outcomes of HIV-positive individuals, identified through HIVST, after entering HIV care, to inform potential users and providers on the benefits of HIVST. The cost of providing HIVST is comparable to standard facility-based HIV testing and counseling (HTC), but the lower yield of positive individuals makes it more costly for identifying those who are HIV-positive.^6^ In contrast to HIVST, facility HTC services are more commonly accessed by those with advanced HIV disease,^4,7^ with individuals needing additional medical care to manage comorbidities.^8,9^ Engaging individuals early within HIV care and treatment through HIVST may yield later cost savings. Improvements in HRQoL among those initiating ART after testing HIV-positive through facility HTC services have been demonstrated^10^; this has yet to be shown for those identified through HIVST. Accurate and contemporaneous understanding of these economic outcomes will be essential to inform policy on scale-up.

We recruited a cohort of adults attending HIV treatment clinics in Blantyre, Malawi, after they had undergone HIVST or facility-based HTC. Our primary aim was to compare the economic costs incurred by health providers and patients and to compare health-related quality of life outcomes for adults diagnosed through HIVST or facility-based HTC.

## METHODS

### Study Design and Participants

We undertook a prospective cohort study in Blantyre, Malawi, between March 2013 and January 2015. We recruited HIV-positive adults identified through either HIVST or facility-based HTC who were participants of a cluster-randomized trial investigating health outcomes of offering HIVST (ISRCTN02004005).^4,5^ Ethical approval was obtained from the College of Medicine Ethics Review Committee, University of Malawi, and the University of Warwick Biomedical Research Ethics Committee. All participants provided informed consent.

The cluster-randomized trial comprised a population of approximately 34,000 residents^4–6^ where adult HIV prevalence was approximately 18%.^11^ Participants in control clusters had access to routine facility-based HTC, and those in intervention clusters were offered HIVST through resident community counselors in addition to facility-based HTC. Participants who self-tested did not have to disclose their HIV test result to community counselors but were offered posttest counseling, advice on where to seek care and a “self-referral card” for HIV clinics. HIVST was provided in the intervention clusters for a 2-year period, starting in February 2012.

We recruited participants from 3 HIV clinics located in the study areas: Queen Elizabeth Central Hospital (QECH), Ndirande Health Centre, and Chilomoni Health Centre. At the start of this study, these clinics had initiated 19,929, 6656, and 4485 individuals onto ART.^12^ Eligible participants were HIV-positive adults (aged ≥18 years) attending for first assessment for ART initiation and resident within trial clusters (verified using global position system-based “Map Book”^13^). Participants who had not accessed either HIVST or facility-based HTC, or who had been assessed for ART initiation or started ART at another location, were excluded.

All care was provided by the routine health system. HIV-positive individuals underwent CD4 count measurements, tuberculosis (TB) screening, provision of cotrimoxazole, and ART adherence counseling. Multiple visits may have been required to complete this assessment. Those who met Malawi national ART eligibility criteria (CD4 count <350 cells/mm^3^ or WHO stage 3 or 4, or breastfeeding or pregnant) were initiated onto ART.

Participants initiated onto ART returned to the HIV clinic at regular intervals for assessment by clinic nurses [or clinical officers (available at all clinics), or doctors (available at QECH only) if unwell]. At clinic visits, ART medication was provided, adherence and response to treatment was assessed, and other clinical problems (eg, TB) managed. Visits varied in frequency, depending on response to ART.

We interviewed participants after each visit to the HIV clinic, and if they were initiated onto ART, they were followed-up for one year. On recruitment, the study team administered structured questionnaires, recording age, sex, marital status, educational attainment, employment status, self-reported income, mode of HIV testing (HIVST, or facility HTC), WHO clinical stage, CD4 count before starting ART, and tracing details. Participants were defined as lost to follow-up if they did not return for scheduled clinic visits and could not be traced.

### Direct Health Provider Costs

After each visit to the HIV clinic, the study team used structured questionnaires to record healthcare resources for each participant, including medical personnel seen, investigations performed, and ART and other medications prescribed. Resources related to hospitalization were not available from participants' HIV clinic records. Primary resource-based costing was undertaken to estimate unit costs for each resource input and consequently total direct health provider costs.^14,15^ Appendix A, http://links.lww.com/QAI/A996 provides a detailed description of the costing process, and Appendix B, http://links.lww.com/QAI/A996 the estimated unit costs estimated for healthcare resources from the primary costing studies.

### Direct Non-medical and Indirect Costs

An interviewer-administered questionnaire was also used after each clinic visit to record participants' direct nonmedical and indirect costs and, where appropriate, costs incurred by family member(s) or carer(s) who accompanied them to clinic. Development, language translations, and pilot testing of questionnaires followed previous procedures.^6^ Direct nonmedical costs included costs of transportation, food, drinks, and other items bought as a consequence of health center visits. For indirect costs, we recorded whether participants or their carers had taken time off work, and multiplied time by self-reported income.^16^ There are no formal payments to access public health services in Malawi.

### Health-Related Quality of Life

The Chichewa EuroQoL EQ-5D-3L^17^ was used to measure HRQoL after each clinic visit. Participants completed both the descriptive EQ-5D-3L system and the accompanying visual analogue scale (VAS).^18^ Responses to the 5 dimensions (mobility; self-care; usual activities; pain; anxiety) of the EQ-5D-3L descriptive system were converted into an EQ-5D utility score using a tariff. Tariff sets have been derived from national surveys of the general population, with a subset of the 243 health states being valued, most commonly using the time trade-off method.^18^ As there is no Malawian EQ-5D tariff, we used the Zimbabwean EQ-5D tariff set to derive an EQ-5D utility score for each study participant at each time point.^19^ The VAS is similar to a thermometer and ranges from 100 (best imaginable health state) to 0 (worst imaginable health state). Participants recorded how good or bad their health was on the day of the clinic visit by drawing a line on the scale.

### Statistical Analysis

Analyses used Stata version 13.1 (Stata Corporation, TX). Costs were converted into 2014 US Dollars and International Dollars.^20,21^ International dollars are hypothetical units of currency that take into account differences in purchasing power across countries, thereby providing a means of comparing cost estimates across jurisdictions. Principal component analysis was used to generate wealth quintiles combining socioeconomic variables, which included 9 household assets and home environment variables.^22^

We undertook multiple imputation using chained equations to impute missing values for cost and HRQoL estimates for participants lost to follow-up.^23^ Comparable to previous studies, our imputation models included mode of HIV testing received, baseline CD4 count, age, sex, and socioeconomic variables.^24,25^ We used predictive mean matching to impute missing values for cost and HRQoL outcomes as they were nonnormally distributed, and to ensure imputed costs were nonnegative.^26^

We estimated the total direct health provider cost, total direct nonmedical and indirect cost, and total societal costs for each study participant. For direct health provider costs, we first estimated total cost for clinic consultations, total costs for investigations, and total costs for treatments. These costs were summed to estimate total direct health provider costs. Health provider costs only included the costs of providing HIV and related medical care at the clinics. The total societal cost was estimated by summing all direct and indirect costs.

We estimated costs for 2 time periods. The first was for the ART assessment period. This included all costs from first attendance to the HIV clinic and continued until the clinic had decided whether a participant was eligible for ART initiation. The second was for the first year on ART and included all costs from the first visit to be initiated onto ART until the participant had been on ART for one year. We estimated mean differences in these costs by mode of HIV testing using bootstrap methods with 500 replications to estimate bias-corrected 95% confidence intervals (CI).^27^ We undertook multivariable analysis to investigate the independent effects of mode of HIV testing on costs. The multivariable model was adjusted for age, sex, and other socio-demographic variables, in addition to baseline CD4 count.^8^ We used generalized linear models (GLM) and ran model diagnostics to determine optimal choices for distributional family and link functions.^28^

For HRQoL assessments, we estimated EQ-5D utility and VAS scores immediately before ART initiation, and for those who initiated ART, after one-year of treatment. We estimated mean differences, and 95% bootstrapped CIs, in HRQoL outcomes by mode of HIV testing received. In addition, we undertook multivariable analysis to investigate the independent effects of mode of HIV testing and baseline CD4 count on the EQ-5D utility scores. The multivariable models were additionally adjusted for age, sex, and other socio-demographic variables. As EQ-5D utility scores are nonnormally distributed, negatively skewed and truncated at 1.0, we evaluated 4 commonly used estimators for our multivariable analyses: ordinary least squares (OLS) regression, tobit regression, fractional logit regression, and censored least absolute deviations (CLAD) regression.^29–31^ We compared mean squared error (MSE) and mean absolute error (MAE) statistics between observed and estimated EQ-5D utility scores to determine the choice of estimator. We also undertook sensitivity analysis using the UK York A1 tariff^32^ to investigate the impact of using an alternative tariff to determine EQ-5D utility scores.

## RESULTS

**FIGURE 1.**
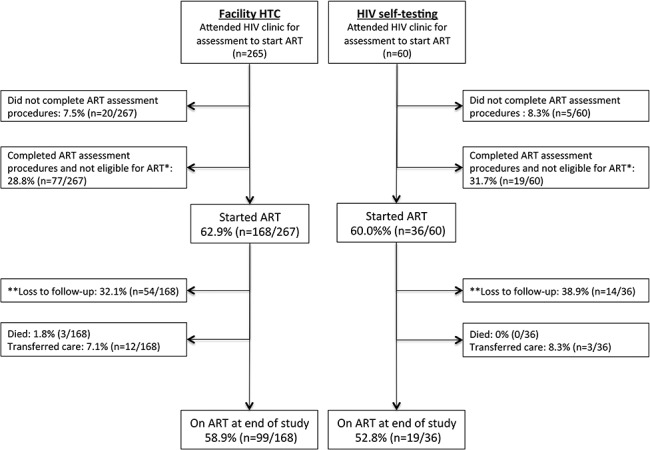
Participant recruitment and follow-up. *Malawi national ART eligibility criteria during study period: CD4 count <350 cells/mm^3^; WHO stage 3 or 4; breastfeeding; or pregnant. **Loss to follow-up from this health economic study.

A total of 325 trial residents attended the HIV clinics for assessment for ART initiation over the study period: 265 after facility-based HTC and 60 after HIVST (Fig. 1). Of the 265 facility-based HTC participants, 20 (7.5%) did not complete ART assessment procedures, 77 (28.8%) completed ART assessment but did not meet Malawian eligibility criteria for initiating ART, and 168 (62.9%) completed ART assessment procedures and initiated ART. Of the 60 HIVST participants, 5 (8.3%) did not complete ART assessment procedures, 19 (31.7%) were not eligible to start ART, and 36 (60.0%) initiated ART. There was no significant difference in the characteristics of ART assessed participants across the 2 groups, except for WHO clinical stage, where there was a higher proportion of missing data for the HIVST group (Table 1).

**TABLE 1.**
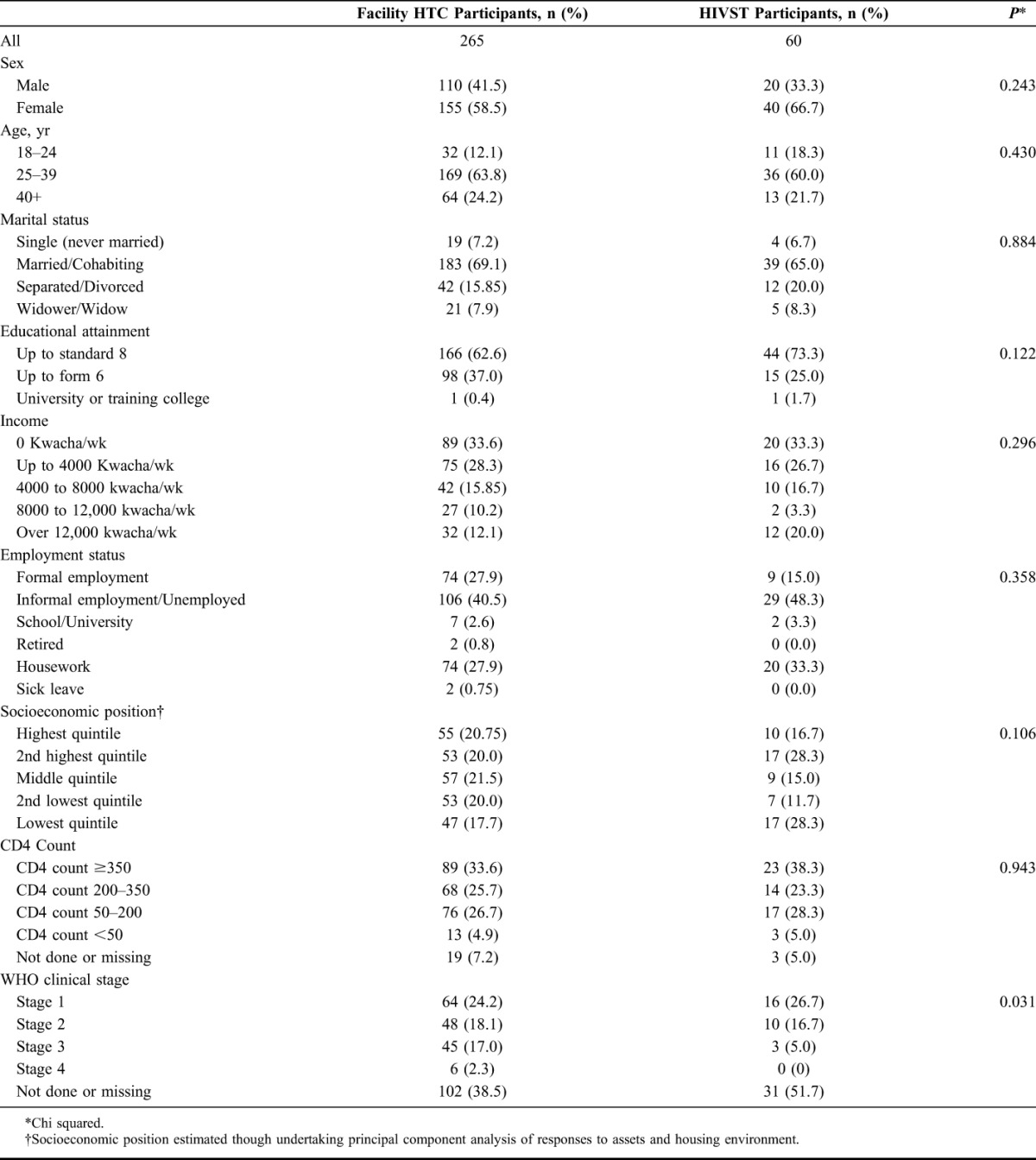
Characteristics of ART Assessed Participants

The mean total health provider costs during the assessment period for ART initiation were US$22.79 for facility HTC participants and US$19.92 for HIVST participants (Table 2). During this period, the mean health provider costs for clinic consultations were US$3.33 (bootstrap 95% CI: US$2.17 to US$4.50) lower for the HIVST group. The mean health provider costs for drug and other medical treatments received were US$0.74 (bootstrap 95% CI: US$0.33 to US$1.16) lower for the HIVST group. The mean health provider costs for investigations performed were not significantly different between the 2 groups. The mean total health provider cost was US$2.87 (bootstrap 95% CI: US$1.01 to US$4.73) lower for the HIVST group. During the assessment period for ART initiation, the mean total direct nonmedical and indirect costs were US$3.31 for facility HTC participants and US$2.65 for HIVST participants. The mean total direct nonmedical and indirect costs were not significantly different between the 2 groups. The mean total societal cost over this period was US$3.54 (bootstrap 95% CI: US$0.37 to US$6.71) lower for the HIVST group.

**TABLE 2.**
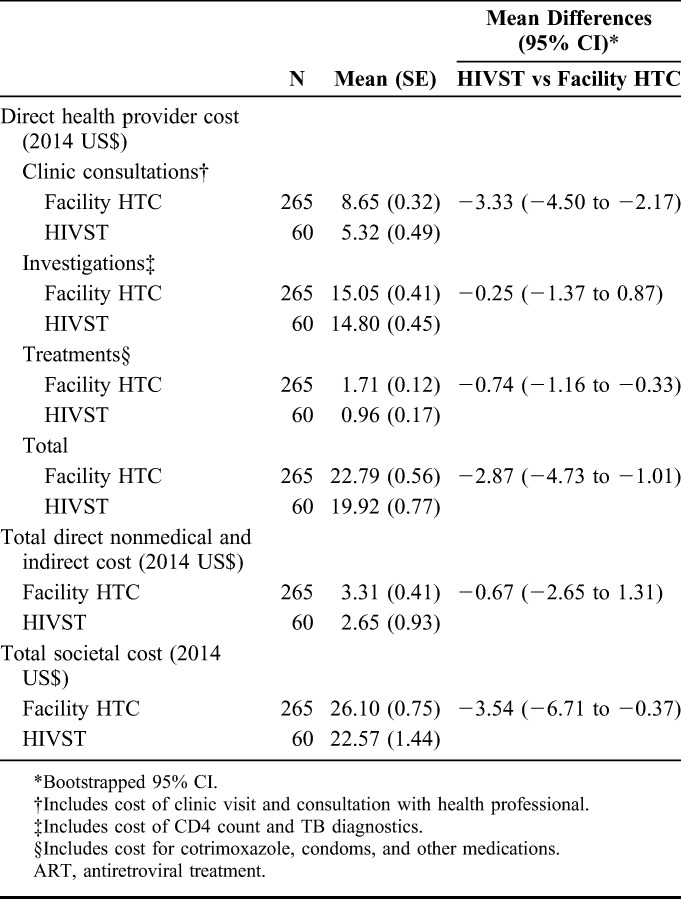
ART Assessment Costs by Mode of HIV Testing (2014 US Dollars)

The mean total health provider costs during the first year following ART initiation were US$168.65 for facility HTC participants and US$164.66 for HIVST participants (Table 3). There were no significant differences in mean health provider costs for clinic consultations, mean health provider costs for treatments and investigations, or for mean total health provider costs between the 2 groups. The mean total direct nonmedical and indirect costs during the first year following ART initiation were US$10.44 for facility HTC participants and US$12.03 for HIVST participants. The mean total direct nonmedical and indirect costs were not significantly different between the 2 groups. The mean total societal costs during the first year following ART initiation were US$178.46 for facility HTC participants and US$177.55 for HIVST participants. The mean total societal costs were not significantly different between the 2 groups.

**TABLE 3.**
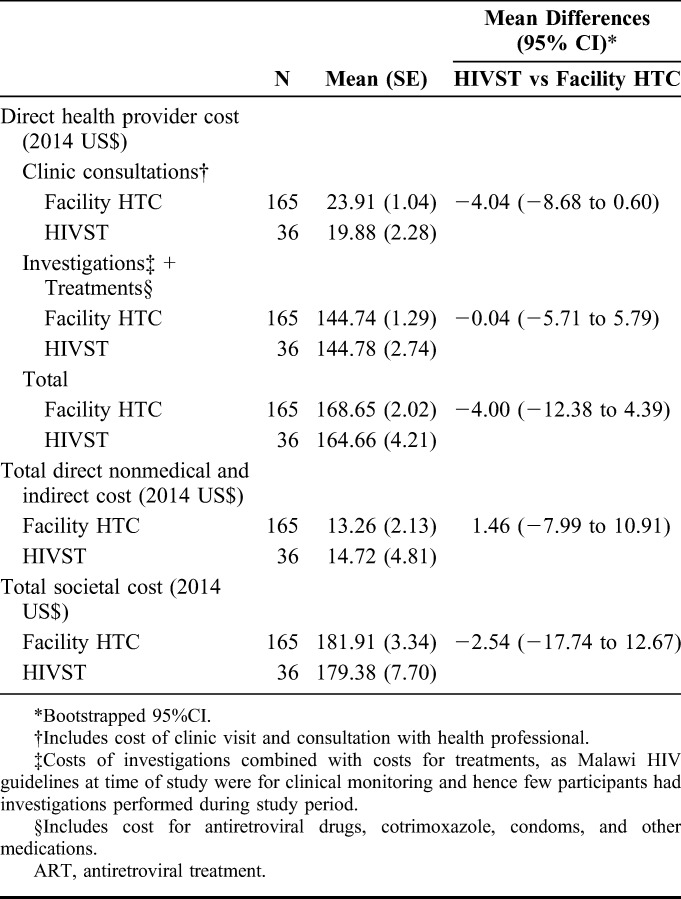
First Year ART Costs by Mode of HIV Testing (2014 US Dollars)

In the multivariable analysis (Table 4), after adjusting for participants' socio-demographic characteristics and CD4 count on ART assessment, the mean total provider cost for ART assessment was US$3.18 (95% CI: US$1.77 to US$4.59) lower for the HIVST group. The mean total societal cost for ART assessment was US$3.86 (95% CI: US$1.64 to US$6.08) lower for the HIVST group. There were no significant differences in mean total provider costs or mean total societal costs during the first year following ART initiation between facility HTC and HIVST participants. Appendix C, http://links.lww.com/QAI/A996 provides the results from the cost analysis in 2014 INT dollars.

**TABLE 4.**
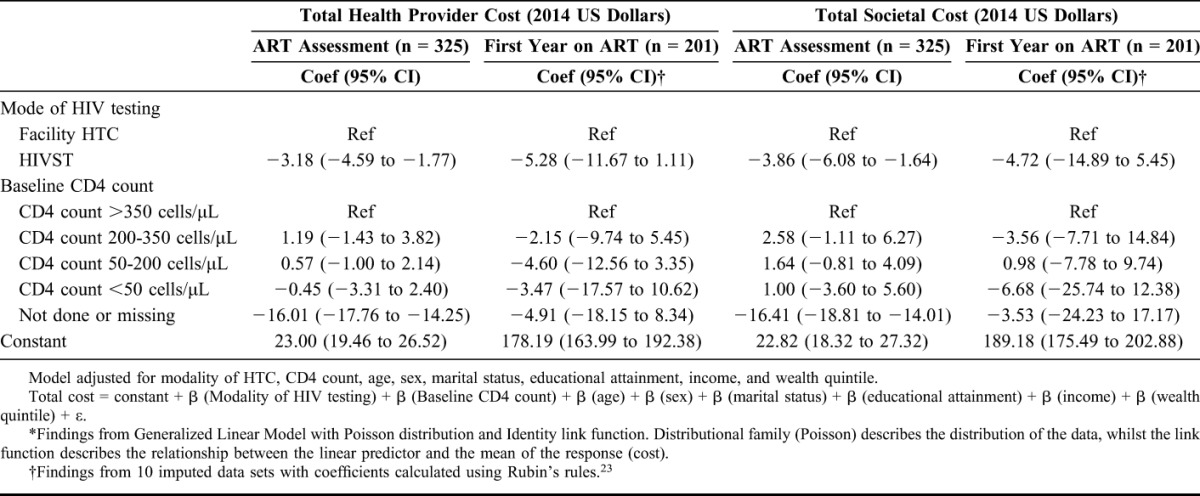
Multivariable Analysis Exploring Relationship Between CD4 Count and Mode of HIV Testing, and ART Assessment and First Year ART Costs (2014 US Dollars)*

The HRQoL outcomes for those who were assessed for ART, immediately before initiation and at 1-year post-ART initiation, and the change in HRQoL scores between these time points, are summarized in Table 5. There were no significant difference in EQ-5D utility and VAS scores immediately before or 1-year post-ART initiation between the 2 groups. Participants who were initiated onto ART experienced improvements in EQ-5D utility and VAS scores. For facility HTC participants who started ART, EQ-5D utility scores increased by 0.129 (SE: 0.011) and VAS scores increased by 9.8 (SE: 1.7). For HIVST participants who started ART, EQ-5D utility scores increased by 0.139 (SE: 0.027) and VAS scores increased by 10.4 (SE: 4.6). There were no significant differences between the 2 groups with regard to the change in EQ-5D utility and VAS scores after ART initiation.

**TABLE 5.**
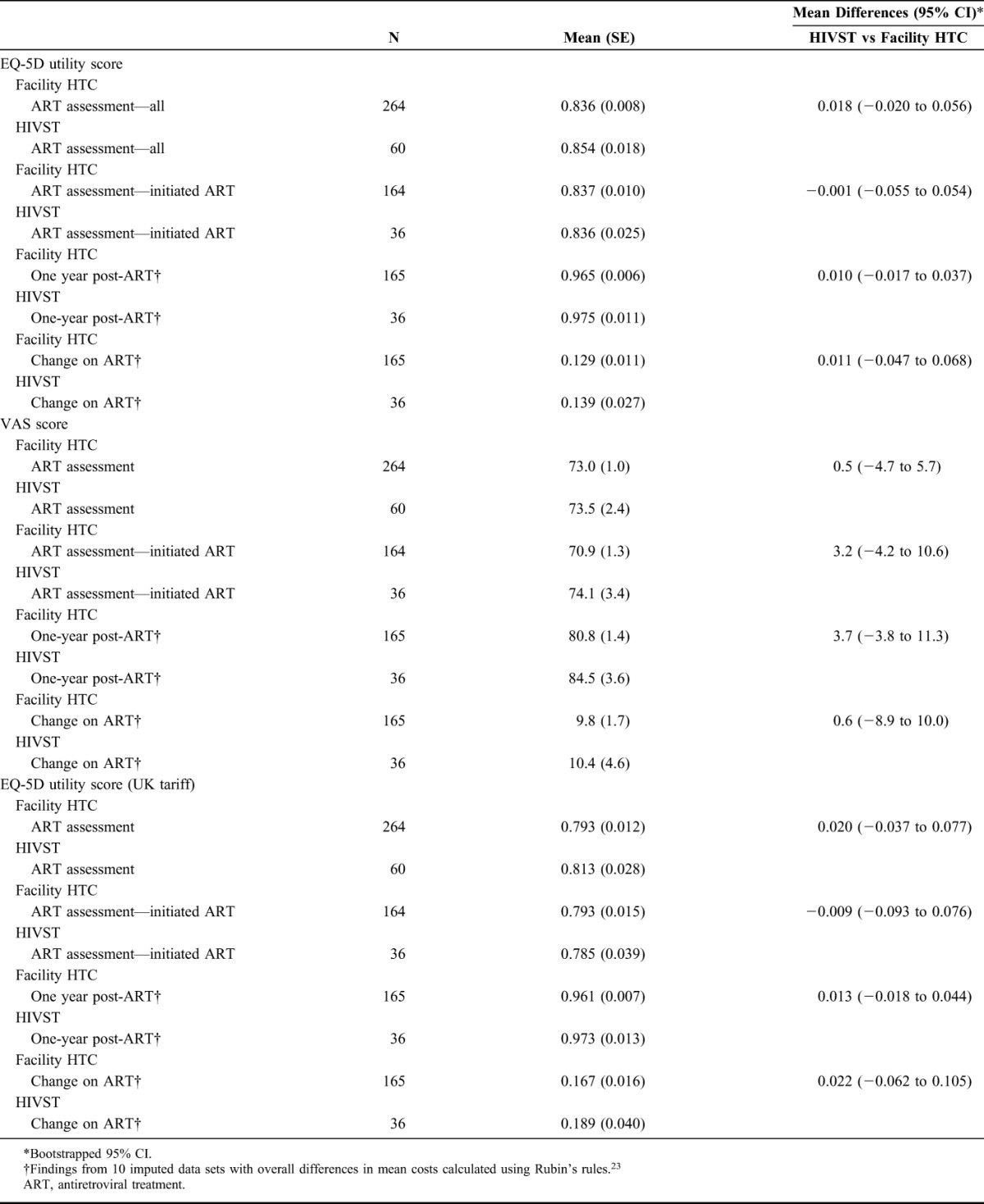
Health-Related Quality of Life Outcomes Immediately Before and 1-Year After ART Initiation by Mode of HIV Testing

In the multivariable analysis (Table 6), the model diagnostics showed that the OLS estimator performed as well or better than the other estimators (Appendix D, http://links.lww.com/QAI/A996). In the fully adjusted OLS model, there was no significant difference in the mean EQ-5D utility score by mode of HIV testing. In the fully adjusted OLS model, the mean EQ-5D utility score was 0.043 (95% CI: 0.008 to 0.079) lower in individuals whose CD4 count was 50–200 cells/μL compared with those whose CD4 count was ≥350 cells/μL on assessment for ART. The mean EQ-5D utility score was 0.230 (95% CI: 0.163 to 0.296) lower in individuals whose CD4 count was below 50 cells/μL compared with those whose CD4 count was ≥350 cells/μL on assessment for ART.

**TABLE 6.**
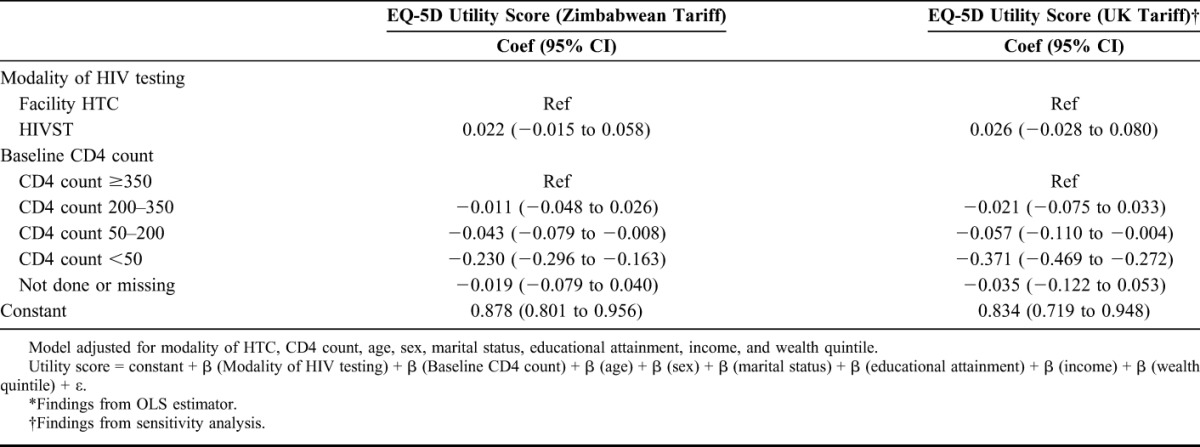
Multivariable Analysis Exploring Relationship Between CD4 Count, Mode of HIV Testing and Pre-ART EQ-5D Utility Score*

## DISCUSSION

The main finding of this study was that the economic costs of providing HIV care and ART to HIV-positive individuals identified through HIVST were comparable to those identified through standard facility-based HTC services. Health-related quality of life was worse among those with lower CD4 counts, with improvements seen after ART initiation, irrespective of mode of HIV testing. These findings emphasize that once HIV self-testers are linked into HIV services, their economic outcomes are comparable to those linked to services after facility-based HTC.

Health provider costs for assessing HIV-positive individuals for ART initiation were lower for HIV self-testers. This difference was because of lower health provider costs associated with clinic consultations and from provision of medical treatments. Additionally, fewer HIV self-testers were clinically assessed as WHO stage 3 or 4. In comparison with community-based HIV testing services, individuals accessing HIV testing at health facilities were often unwell for other reasons (eg, TB) or have more advanced HIV clinical disease.^33^ These individuals may need medical care for management for these other problems or for investigation to exclude HIV associated illnesses before initiating ART. Although the cost savings demonstrated are small at the individual-level, at the population-level, these could be significant with increasing availability of HIVST.

We estimated the annual health provider cost of managing a patient on ART to be approximately 2014 US$170, comparable to previous estimates for Malawi (US$136 per person per year in 2011).^34^ Health provider and societal costs were not affected by modality of HIV testing before entering HIV care services. Malawi has followed a public health approach to scaling-up its HIV treatment services with less reliance on diagnostic tests for clinical assessment, and therefore the majority of individuals use comparable levels of healthcare resources.^35^ We did not find differences in healthcare utilization between the 2 groups. Although it is reassuring that these costs were comparable, the findings highlight opportunities to explore how HIV treatment should be provided as we move toward universal access to ART.^36^

The study demonstrates the relatively high costs incurred by patients when accessing HIV care. Individuals incurred a cost of approximately US$3 during their assessment for ART eligibility and US$13 during the first year following ART initiation. The majority of Malawians live on less than $2 a day.^37^ Antiretroviral therapy is provided free, but those accessing care incur costs of transport or because of taking time off work to attend clinics.^38^ These costs can also have a negative impact on adherence to therapy.^39,40^ ART can be effectively provided in people's homes through community distribution models.^5,41^ Further work is needed to explore the risks and benefits of home provision of treatment.

HRQoL as measured by the EQ-5D has been shown to be responsive to change among HIV-positive patients in high-income settings,^42^ but few studies have used this measure in sub-Saharan African settings.^10^ The EQ-5D utility score provides an objective assessment of HRQoL for cost-utility analysis, with the VAS scores reflecting respondents' own assessments of their HRQoL. We found that EQ-5D utility scores to be significantly associated with an HIV-positive individual's CD4 count, with improvements after initiation of ART. Participants also reported higher VAS scores after ART initiation. The findings support the beneficial impact of ART on both quality and quantity of life and illustrate the importance of reaching those not in care before their disease advances. The mode of HIV testing had no independent impact on HRQoL outcomes.

This study is not without its limitations. The numbers recruited into the study were small, and many were lost to follow-up. Although we undertook multiple imputation to account for this, our findings may be limited because those lost to follow-up are potentially a sicker population, with poorer HRQoL, and, had they remained in care, higher healthcare resource use. We were not able to include healthcare resources used as a result of hospitalization because there was no routine medical record keeping or linking of records between community, outpatient, and inpatient services. Furthermore, some of the unit costs estimated for the healthcare resource inputs, for example costs of consultations with a healthcare worker, represent average costs for average reported duration of consultations. These information system issues reduced our ability to detect differences in economic outcomes, but are unlikely to bias our findings.

A further limitation is that the EQ-5D tool only evaluates HRQoL across 5 health dimensions and may therefore not capture all relevant aspects of HRQoL. The lack of a Malawian tariff led us to use the Zimbabwean tariff to derive EQ-5D-3L utility scores. However, the EQ-5D tool is widely used for health economic analyses, and it is accepted practice to use tariffs from another country where none exists for the country of interest provided the 2 populations would value health comparably.^15^ A final study limitation is that the recent change in ART initiation guidelines^36^ means that we are unable to comment on the economic outcomes of those who would in the future start treatment with early HIV disease.

In conclusion, we found that once HIV self-testers link into HIV treatment services, the costs of providing HIV care and improvements in HRQoL from ART are no different to those identified through facility-based HTC. The findings add to the growing literature supporting the scale-up of HIVST in the region. Full economic evaluations are needed to explore whether implementing HIVST is cost-effective. Our assessments of economic costs and preference-based HRQoL outcomes can help inform such analyses.
